# Supplementary material for: Measuring Mobility in Chromatin by Intensity-Sorted FCS
Source: Biophys J. 2019 Feb 14;116(6):987–99. doi: 10.1016/j.bpj.2019.02.003 (PMC6428914; doi:10.1016/j.bpj.2019.02.003)
Supplement: Document S1. Figs. S1–S8 [file mmc1.pdf]

Biophysical Journal, Volume *116*

Supplemental Information

Measuring Mobility in Chromatin by Intensity-Sorted FCS

Melody Di Bona, Michael A. Mancini, Davide Mazza, Giuseppe Vicidomini, Alberto Diaspro, and Luca Lanzanò

Supplementary Figures

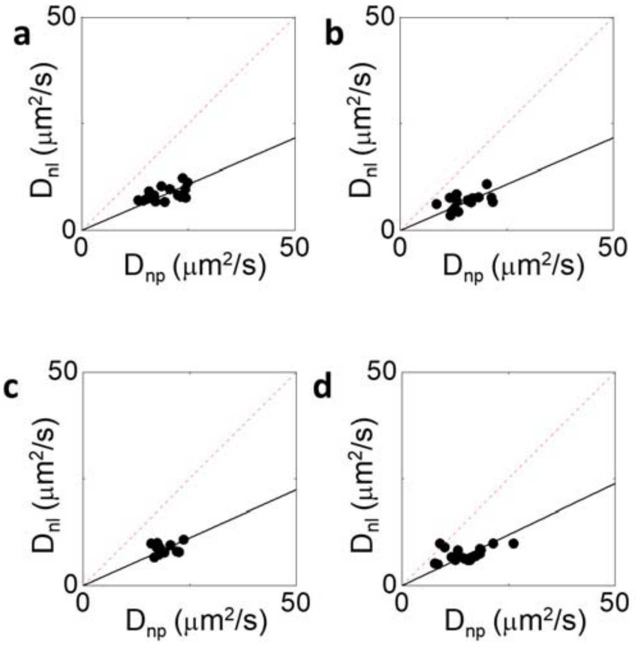

Fig S1. Results of the additional experiments of untagged GFP diffusion in the nucleoplasm (np, x-axis) and nucleolus (nl, y-axis). (a) Diffusion coefficients calculated on 15 cells with a slope of 0.43. (b) Diffusion coefficients calculated on 15 cells with a slope of 0.43. (c) Diffusion coefficients retrieved from measurements done on 11 cells with a slope of 0.45. (d) Diffusion coefficients calculated on 20 cells, with a slope of 0.48.

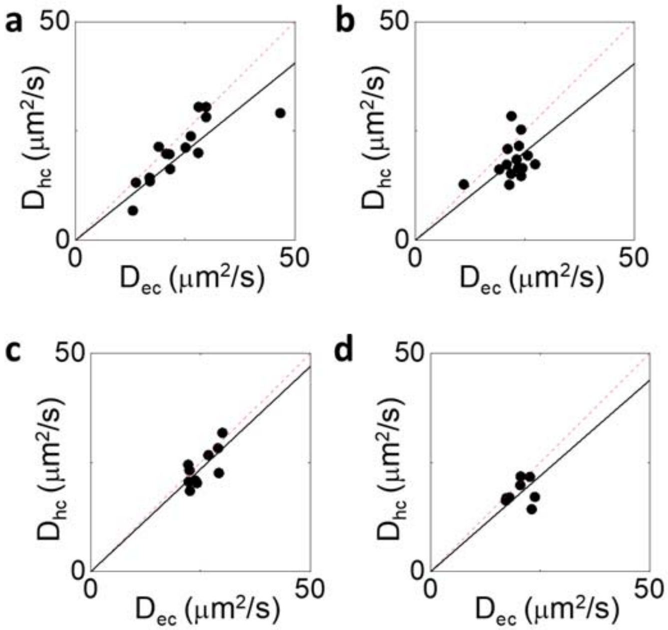

Figure S2. Results of the additional experiments of untagged GFP diffusion performed on the nucleoplasm of cells marked with Hoechst. For each experiment, is shown the scatter plot of the diffusion coefficient measured on heterochromatin (y-axis) versus the diffusion coefficient measured on euchromatin (x-axis) on the same cell, along with the corresponding linear fit through the origin. (a) Diffusion coefficients calculated on 16 cells, with a slope of 0.81 (b) Measurements done on 15 cells, with a slope of 0.81. (c) Diffusion coefficients measured on 10 cells, with a slope of 0.94. (d) Diffusion coefficients retrieved on 8 cells, with a slope of 0.88.

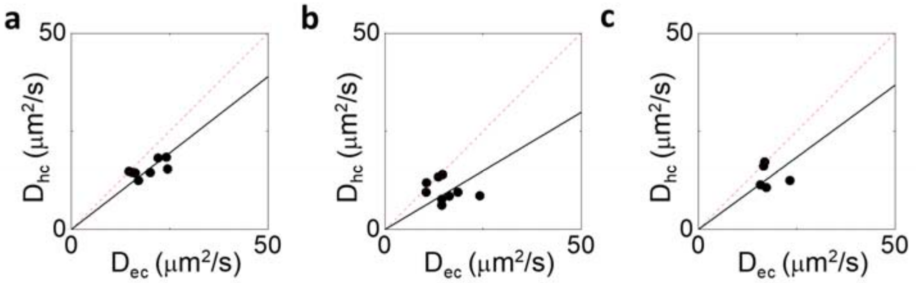

Figure S3. Results of the additional experiments of untagged GFP diffusion in euchromatin versus perinucleolar heterochromatin. For each experiment, is shown the scatter plot of the diffusion coefficients measured on perinucleolar heterochromatin (y-axis) versus euchromatin (x-axis) along with the corresponding linear fit through the origin. (a) Diffusion coefficients calculated on 8 cells, with a slope of 0.78. (b) Measurements done on 9 cells, with the linear fit having a slope of 0.6. (c) Diffusion coefficients measured on 5 cells, with a slope of 0.73.

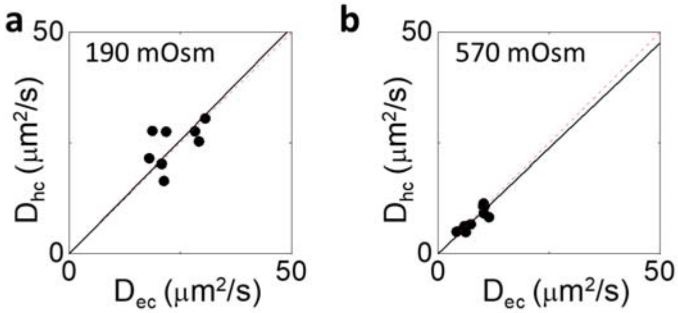

Figure S4. Results of the additional measurements of untagged GFP diffusion in eu- vs hetero-chromatin after osmotic treatments. For each experiment, is shown the scatter plot of the diffusion coefficients measured on heterochromatin (y-axis) versus euchromatin (x-axis) along with the corresponding linear fit through the origin. (a) Diffusion coefficients calculated on 8 cells treated with an hypo-osmolar solution, with a slope of 1. (b) Diffusion coefficients measured on 10 cells treated with a hyperosmolar solution, with a slope of 0.95.

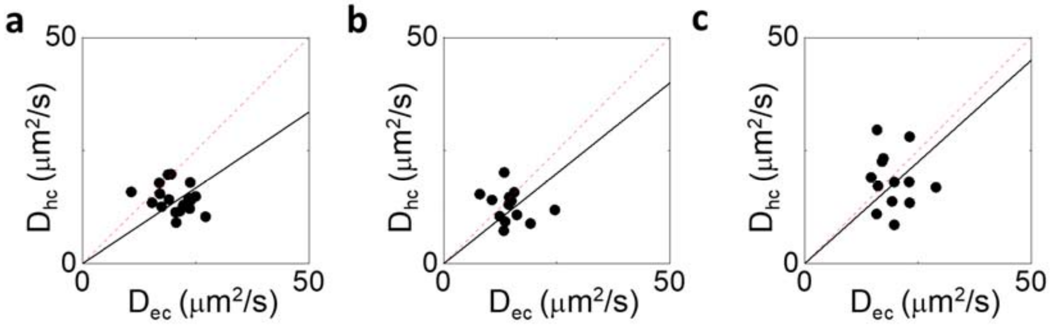

Figure S5. Repetition of measurement of the untagged GFP diffusion coefficient after the ATP-depletion treatment. For each experiment, is shown the scatter plot of the diffusion coefficients measured on heterochromatin (y-axis) versus euchromatin (x-axis) along with the corresponding linear fit through the origin. (a) Diffusion coefficients calculated on 18 cells with a slope of 0.67. (b) Measurement done on 13 cells with a slope of 0.8. (c) Diffusion coefficients measured on 13 cells with a slope of 0.9.

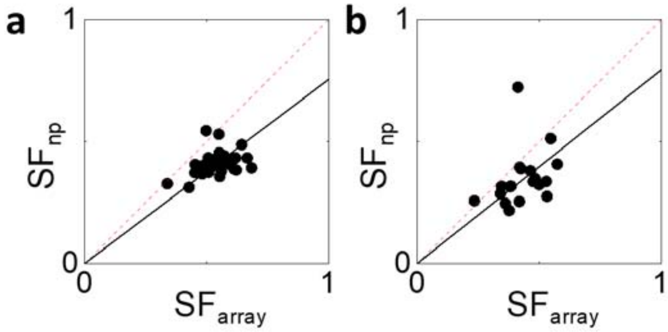

Figure S6. Repetition of measurements done on the mobility of the Estrogen Receptor inside (x-axis) and outside (y-axis) a nuclear array of prolactin genes, analyzed with a two diffusion component model. The slow diffusing fraction of protein calculated on array is plotted against the fraction retrieved from the nucleoplasm. (a) Slow diffusing ER fraction calculated on 30 cells with a slope of 0.75. The two diffusion coefficients retrieved are 3.1$\mu m^2/s$ and 0.1 $\mu m^2/s$. (b) Slow diffusing ER fraction calculated on 19 cells with a slope of 0.79. The two diffusion coefficients retrieved are 3 $\mu m^2/s$ and 0.1 $\mu m^2/s$.

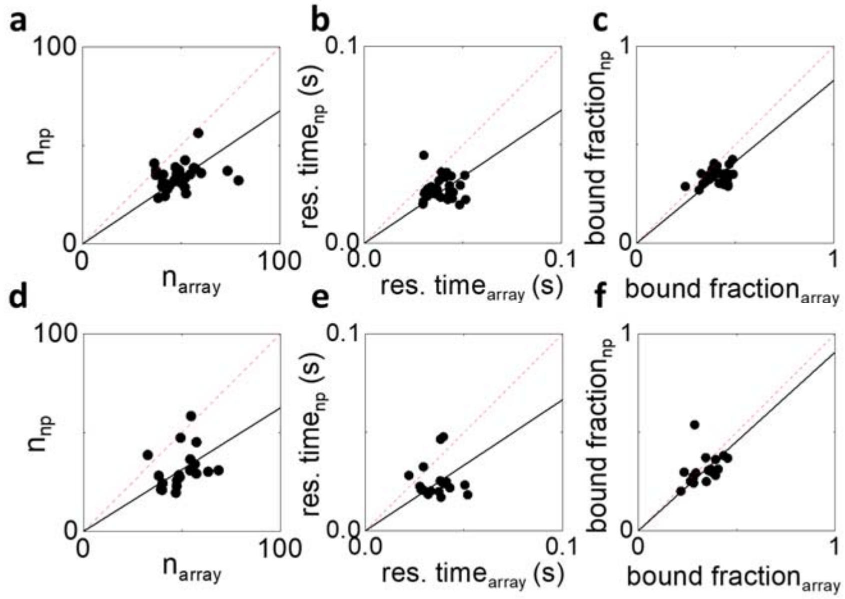

Figure S7. Repetition of measurements done on the mobility of the Estrogen Receptor inside (x-axis) and outside (y-axis) the array of prolactin genes, analyzed with the Full Model. The measurements are performed on 30 (a-c), and 18 cells (d-f) plotting the number of molecules (n) found in the two regions (a, d; slopes 0.68 and 0.63, respectively), the average residence time of the ER on the binding site (b, e; slopes 0.68 and 0.67, respectively) and the fraction of bound protein (c, f; slopes 0.83 and 0.91, respectively).

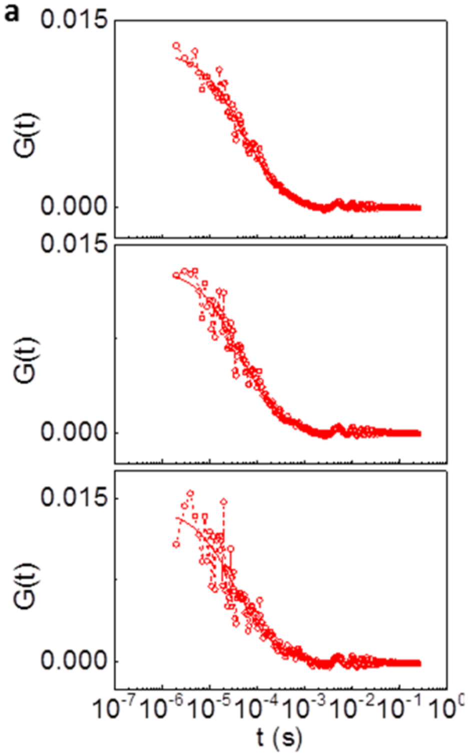

Figure S8. Calibration of the detection volume in a GFP solution. The diffusion coefficient of GFP in solution is fixed to 90μm^2/s, and the fit is performed in order to retrieve the effective volumes for each mathematical filter used in the analysis. The ACFs are calculated using, from top to bottom, increasingly smaller number of photons: the fit gives a value of the effective volume size of 137, 126 and 114 nm, from top to bottom.
